# Higher Dimensional Rotating Black Hole Solutions in Quadratic *f*(*R*) Gravitational Theory and the Conserved Quantities

**DOI:** 10.3390/e23030358

**Published:** 2021-03-17

**Authors:** Gamal G. L. Nashed, Kazuharu Bamba

**Affiliations:** 1 Centre for Theoretical Physics, The British University in Egypt, P.O. Box 43, El Sherouk City, Cairo 11837, Egypt; nashed@bue.edu.eg; 2 Division of Human Support System, Faculty of Symbiotic Systems Science, Fukushima University, Fukushima 960-1296, Japan

**Keywords:** black hole solutions, modified gravity, thermodynamics of black holes

## Abstract

We explore the quadratic form of the $f\left(R\right)=R+bR^{2}$ gravitational theory to derive rotating *N*-dimensions black hole solutions with $a_{i},i\geq 1$ rotation parameters. Here, *R* is the Ricci scalar and *b* is the dimensional parameter. We assumed that the *N*-dimensional spacetime is static and it has flat horizons with a zero curvature boundary. We investigated the physics of black holes by calculating the relations of physical quantities such as the horizon radius and mass. We also demonstrate that, in the four-dimensional case, the higher-order curvature does not contribute to the black hole, i.e., black hole does not depend on the dimensional parameter *b*, whereas, in the case of N>4, it depends on parameter *b*, owing to the contribution of the correction $R^{2}$ term. We analyze the conserved quantities, energy, and angular-momentum, of black hole solutions by applying the relocalization method. Additionally, we calculate the thermodynamic quantities, such as temperature and entropy, and examine the stability of black hole solutions locally and show that they have thermodynamic stability. Moreover, the calculations of entropy put a constraint on the parameter *b* to be $b<\frac{1}{16\Lambda }$ to obtain a positive entropy.

## 1. Introduction

Einstein’s general theory of relativity (GR) does not provide scientists with an explanation that can support the discovery of the accelerated expansion of our universe that has been established 20-years ago by various observations [1,2,3,4,5,6,7,8,9,10,11,12,13,14,15,16,17,18,19]. Thus, scientists have developed other theories that can support this expansion rate. Among these theories, there is one in which we add a cosmological constant to the field equations of GR. The output model of this theory becomes dominated by this constant, which can explain the accelerated expansion. In the literature, this model is known as Λ cold dark matter (ΛCDM). ΛCDM has a contradiction between gravity and quantum field theory [20]. This leads scientists to modify the structure of GR, either within Riemannian geometry [21] or while using another geometry.

Among the modified theories that used other Riemannian geometry is the f(T) theory, where *T* is the torsion scalar in teleparallel gravity. This theory has second-order differential equations [22,23,24], which makes it easy to analyze its physics. f(T) has been used in the domain of cosmology [25,26,27,28,29] and in the solar system [30,31,32,33]. The other modified theories that used Riemannian geometry are as follows:

i—String theory, which is one of the most possible candidates for the quantum theory for gravitation [34].

ii—Lovelock theory, which is the natural generalization of Einstein’s GR to higher dimensions [35].

iii—Brans–Dicke theory, whose interaction is considered by GR tensor and scalar field [36].

iv—f(R) theory, which we focus on in this study [37,38] (for recent reviews on the dark energy problem and modified gravity theories to explain the late-time cosmic acceleration and inflation in the early universe, see, for instance, [39,40,41,42,43,44,45]).

f(R) theory has several successful applications in the domain of cosmology [46,47,48,49,50]. However, it should be associated with other tests to achieve the success of GR in the solar system [51]. f(R) is a modification of Einstein’s GR and it is considered to be a novel geometrodynamical theory with degrees of freedom in the field equations of gravitation [24,52,53,54,55]. The action integral of this theory contains an appropriate function of the Ricci scalar *R*, and the field equations are of the fourth-order. The lower order of the field equations provides field equations of Einstein’s GR, which are of the second order. A coincidence between f(R) and other modified gravitational theories through different frames can be found in [56].

A viable inflationary model in gravity that considered quantum corrections and included $R+R^{2}$ gravity as a particular case was derived in [57]. The true forms of scalar and tensor perturbations created through inflation in such a model are discussed in [58]. The f(R) theory can describe the inflationary stage and dark energy-dominated stage, in which the late-time cosmic acceleration is realized [59,60,61]. A static spherically symmetric solution is discussed in [62,63,64], whereas studies on gravitational collapse can be found in [65,66]. Several black holes are derived in the framework of f(R) [67,68,69,70,71,72,73,74,75,76,77,78] and their physical consequences are discussed in [79,80,81,82].

The characteristics of gravitational theories in more than four dimensions have been studied more widely [83]. The significant motivation of these studies is to identify the relation between black holes and fundamental theories, such as string theory, besides the consideration of the large extra dimensions with models of the TeV-scale gravity. The particular higher-dimensional solutions in classical GR have been found in the extensions to any n>4 of the Schwarzschild and Reissner-Nordström black holes that were derived by Tangherlini [84] and that of the Kerr black hole solution analyzed by Myers and Perry [85]. However, new investigations have indicated that, even at the classical level, higher-dimensional gravity theories have much richer dynamics for n>4. The non-uniqueness of asymptotically-flat rotating black holes is one of the most important features. For instance, for GR in a five-dimensional vacuum, $S^{1}\times S^{2}$ rotating black ring solutions have been acquired explicitly [86]. This object can have the same mass and spin as those of the $S^{3}$ black holes suggested in Ref. [85]. Such a violation will be continuously infinite for the rings with magnetic dipole charge [87].

One has to analyze black hole solutions to provide a good probe of the f(R) gravitational theory. The exact solutions for f(R) are a hot topic, and there are several studies on the topic, starting from three-dimensions [88] to *N*-dimensions [89,90]. Analytic solutions that describe rotating black holes are derived in [90,91,92]. The main purpose of this work is to derive solutions for the *N*-dimension black hole with flat or cylindrical horizons in the framework of $f\left(R\right)=R+bR^{2}$. To achieve this, we used a general N-dimension metric that possesses a k−dimension Euclidean metric and derived a static black hole solution in diverse dimensions. Using a coordinate transformation, we successfully derive a stationary rotating black hole solution for $f\left(R\right)=R+bR^{2}$. The physics of these black holes is investigated by calculating conserved quantities and studying thermodynamic quantities, such as Hawking temperature, entropy, and heat capacity. The influence of higher-order curvature corrections on the existence of relativistic compact objects in modified gravity has been argued [93,94].

This paper is organized, as follows: in Section 2, we provide the basics of the f(R) gravitational theory and N-dimension spacetime with one unknown function is applied those to the quadratic form of f(R) field equations. Additionally, in Section 2, black hole solutions are derived for two different cases, i.e., four-dimensional case and N>4. In Section 3, we apply a coordinate transformation to the black hole solutions that are derived in Section 2 and obtain rotating black hole solutions that satisfy the field equations of the quadratic form of the f(R) theory. In Section 4, we calculate the conserved quantities of the rotating solutions using the Komar method and obtain divergent quantities. In Section 5, we use the regularized method and redo the calculations of the conservation and obtain finite quantities for rotating black holes. Finally, in Section 6, we discuss the stability of black hole solutions locally and then explain that the derived solutions are stable from the viewpoint of thermodynamics. In Section 7, we present our conclusions and discussion.

## 2. Basics of f(R) Gravitational Theory

We consider a gravitational field with a cosmological constant. The action of this field is given by [64,72,95,96,97]

(1) $$S:=\frac{1}{2\chi }\int d^{N}x\sqrt{-g}\left(f\left(R\right)-2\Lambda \right),$$

where Λ is the cosmological constant and χ is the *N*-dimension gravity constant that is represented by $\chi =2\left(N-3\right)\Omega_{N-1}G_{N}$, where $G_{N}$ is the gravitation constant of Newton in *N*-dimensions. In this study, $\Omega_{N-1}$ shows the volume for the (N−1)-dimensional unit sphere. It is given by [64,72,95,96]

(2) $$\Omega_{N-1}=\frac{2\pi^{(N-1)/2}}{\Gamma ((N-1)/2)},$$

where Γ is the gamma function.

By varying Equation (1) with respect to the metric tensor $g_{\mu \nu }$, the field equations for f(R) can be derived in the following form [98,99]:

(3) $$E_{\mu \nu }\equiv R_{\mu \nu }f_{R}-\frac{1}{2}g_{\mu \nu }f\left(R\right)-\frac{1}{2}g_{\mu \nu }\Lambda +g_{\mu \nu }□f_{R}-\nabla_{\mu }\nabla_{\nu }f_{R}-2\kappa T_{\mu \nu }=0,$$

where $R_{\mu \nu }$ is the Ricci tensor that is given by

(4) $$R_{\mu \nu }=R^{\rho }{}_{\mu \rho \nu }=\partial_{\rho }\Gamma^{\rho }{}_{\mu \nu }-\partial_{\nu }\Gamma^{\rho }{}_{\mu \rho }+\Gamma^{\rho }{}_{\rho \beta }\Gamma^{\beta }{}_{\mu \nu }-\Gamma^{\rho }{}_{\nu \beta }\Gamma^{\beta }{}_{\mu \rho }=2\Gamma^{\rho }{}_{\mu [\nu ,\rho ]}+2\Gamma^{\rho }{}_{\beta [\rho }\Gamma^{\beta }{}_{\nu ]\mu },$$

and $\Gamma^{\rho }{}_{\mu \nu }$ is the Christoffel symbols of the second kind and the square brackets mean skew-symmetrization. The D’Alembert operator □ is defined as $□=\nabla_{\alpha }\nabla^{\alpha }$, where $\nabla_{\alpha }V^{\beta }$ is the covariant derivatives in terms of the vector $V^{\beta }$, $f_{R}=\frac{df(R)}{dR}$ and $T_{\mu \nu }$ is the energy-momentum tensor. The trace of field Equation (3), in the vacuum case, gives

(5) $$Rf_{R}-\frac{N}{2}f\left(R\right)-8\Lambda +3□f_{R}=0.$$

Equation (5) with f(R)=R gives R=−8Λ.

We will apply the field Equation (3) to the following metric:

(6) $$ds^{2}=-h\left(r\right)dt^{2}+\frac{1}{h(r)}dr^{2}+r^{2}\left(\sum_{i=1}^{\ell }d\phi_{i}^{2}+\sum_{k=1}^{N-\ell -2}dz_{k}{}^{2}\right).$$

Here, 0≤r<∞, −∞<t<∞, $0\leq \phi_{\ell }<2\pi$, $-\infty <z_{k}<\infty$, and h(r) (in this study, we consider spacetime (6) with one unknown function only to make the calculations more applicable. This constraint makes the spacetime not affected by the parameter *b* in four dimensions because of the non-contribution of the $R^{2}$ term. To let the spacetime be affected by the parameter *b*, in the four-dimensional case, we must consider the charged form of the field in Equation (3) [72]) is an unknown function in terms of the radial coordinate *r*. Using Equation (6) we obtain the Ricci scalar, as

(7) $$R=-\frac{r^{2}h^{\prime \prime }+2\left(N-2\right)rh^{\prime }+\left(N-2\right)\left(N-3\right)h}{r^{2}},$$

where $h^{\prime }=\frac{dh(r)}{dr}$ and $h^{\prime \prime }=\frac{d^{2}h\left(r\right)}{dr^{2}}$. The non-zero components of the f(R) field equations, Equations (3), for $f\left(R\right)=R+bR^{2}$, where *b* is a dimension parameter, and $T_{\mu }{}^{\nu }=0$ takes the form (the detailed calculations of the Ricci curvature tensor are given in Appendix B)

(8) $$\begin{array}{ccc} & & E_{t}{}^{t}=\frac{1}{2r^{4}}(br^{3}\left\{2h^{\prime \prime \prime }\left[rh^{\prime }+6h\left(N-2\right)\right]+4rhh^{⁗}-rh^{\prime \prime }{}^{2}\right\}+2\left(N-2\right)r^{2}bh^{\prime \prime }\left(2\left[3N-11\right]h+rh^{\prime }\right)+2\left(N^{2}-7N+10\right)br^{2}h^{\prime }{}^{2}\\ & & -h^{\prime }\left[\left(N-2\right)r^{3}-2brh\left(N-2\right)\left(3N^{2}-29N+64\right)\right]-h\left(N^{2}-5N+6\right)r^{2}+2b\left(N^{2}-N-2\right)h^{2}+4r^{4}\Lambda )=0,\\ & & E_{r}{}^{r}=\frac{1}{2r^{4}}(br^{3}\left\{2h^{\prime \prime \prime }\left[rh^{\prime }+2h\left(N-2\right)\right]-rh^{\prime \prime }{}^{2}\right\}+2\left(N-2\right)r^{2}bh^{\prime \prime }\left(4\left[N-2\right]h+rh^{\prime }\right)+2\left(N^{2}-7N+10\right)br^{2}h^{\prime }{}^{2}\\ & & -h^{\prime }\left[\left(N-2\right)r^{3}+2\left\{4\left(N-2\right)-3\left(N-4\right)\left(N^{2}-5N+6\right)\right\}brh\right]-\left(N-2\right)\left(N-3\right)\left[hr^{2}-b\left\{N^{2}-13N+22\right\}h^{2}\right]+4r^{4}\Lambda )=0,\\ & & E_{\phi_{1}{}^{\phi_{1}}}=E_{\phi_{2}{}^{\phi_{2}}}\cdots E_{\phi_{N-\ell -2}{}^{\phi_{N-\ell -2}}=E_{z_{1}{}^{z_{1}}}=E_{z_{2}{}^{z_{2}}}=\cdots E_{z_{N-2}{}^{z_{N-2}}}}=\frac{1}{2r^{4}}(br^{3}\left\{4h^{\prime \prime \prime }\left[rh^{\prime }+h\left(3N-7\right)\right]+4rhh^{⁗}+rh^{\prime \prime }{}^{2}\right\}\\ & & -r^{2}h^{\prime \prime }\left[r^{2}-2b\left\{2\left(3N-7\right)rh^{\prime }-\left[2\left(N-2\right)-\left(N-4\right)\left(7N-15\right)\right]h\right\}\right]+4\left(N-2\right)\left[2N-9\right]br^{2}h^{\prime }{}^{2}-\left(N-3\right)\left(N-4\right)r^{2}h+4r^{4}\Lambda \\ & & -h^{\prime }\left(2\left(N-3\right)r^{3}+8\left(N-2\right)\left\{2\left(N-3\right)-\left(N-4\right)\left(N-5\right)\right\}brh\right)-2b\left(N-6\right)\left[\left(N-3\right)\left(2N-1\right)-2\left(N-4\right)\left(N-5\right)\right]h^{2})=0. \end{array}$$

The abovementioned system cannot have a general solution because of the appearance of the term (N-4). Therefore, we deal with it in two separate cases. The first case is the four-dimension one, in which the solution for the abovementioned system is expressed as

(9) $$\begin{array}{c} h\left(r\right)=\frac{2r^{2}\Lambda }{3}+\frac{c_{1}}{r}. \end{array}$$

Equation (9) shows that higher curvature has no effect, i.e., solution (9) is identical to GR. The second case is the one in which **N>4**, and its solution takes the form

(10) $$\begin{array}{ccc} & & h\left(r\right)=\frac{\left(N-2\right)r^{2}\left[1+\sqrt{1-\frac{16N(N-4)b\Lambda }{{\left(N-2\right)}^{2}}}\right]}{2N(N-1)(N-4)b}+\frac{c_{2}}{r^{N-3}}\equiv r^{2}\Lambda_{eff}+\frac{c_{2}}{r^{N-3}},\\ & & where\;\Lambda_{eff}=\frac{\left(N-2\right)\left[1+\sqrt{1-\frac{16N(N-4)b\Lambda }{{\left(N-2\right)}^{2}}}\right]}{2N(N-1)(N-4)b}. \end{array}$$

where $c_{1}$ and $c_{2}$ are the integration constants. Equation (10) shows how the solution is affected by the dimension parameter *b*. Additionally, Equation (10) informs us that the parameter *b* should not be equal to 0. In the case where we set the explicit cosmological constant Λ=0 in Equation (10), we obtain

(11) $$\begin{array}{c} \Lambda_{eff}=\frac{\left(N-2\right)}{2N(N-1)(N-4)b}. \end{array}$$

which shows that the parameter *b* is an effective cosmological constant [97,100]. Hence, the metric potential (10) is new and it cannot be reduced to the GR metric when the dimensional parameter b=0. The metric spacetimes of solutions (9) and (10) have the form

(12) $$\begin{array}{ccc} ds_{1}{}^{2} & = & -\left\{\frac{2r^{2}\Lambda }{3}+\frac{c_{1}}{r}\right\}dt^{2}+{\left\{\frac{2r^{2}\Lambda }{3}+\frac{c_{1}}{r}\right\}}^{-1}dr^{2}+r^{2}\left(d\phi_{1}^{2}+dz_{1}^{2}\right)\;,\;\;\;N=4,\\ ds_{2}{}^{2} & = & -\left\{r^{2}\Lambda_{eff}+\frac{c_{2}}{r^{N-3}}\right\}dt^{2}+{\left\{r^{2}\Lambda_{eff}+\frac{c_{2}}{r^{N-3}}\right\}}^{-1}dr^{2}+r^{2}\left(\sum_{i=2}^{n}d\phi_{i}^{2}+\sum_{k=2}^{N-n-2}dz_{k}dz_{k}\right)\;,\;N>4. \end{array}$$

We stress the fact that solution (10) is new for N>4, because it contains the dimensional parameter *b*, and it cannot reduce to GR, because *b*, in this case, is not allowed to take zero value.

The asymptote of Equation (12) behaves as AdS/dS. We must stress the fact that the disappearance of the dimensional parameter *b* in the four-dimensional case is because we deal with a static black hole, however, if we study a charged black hole, this parameter should appear in the four-dimensional case. Moreover, if we change the four-dimensional case to the Einstein frame, the black hole, in that case will depend on the parameter *b*, because the conformal factor $\sigma^{2}=f_{R}=1-16b\Lambda$ [60,101,102] and the metric will change, owing to this transformation, as [60]

(13) $$\begin{array}{c} g_{\mu \nu }\to {\bar{g}}_{{\mu \nu }_{Ein}}\left(x\right)=\sigma^{2}\left(x\right)g_{{\mu \nu }_{Jor}}\left(x\right), \end{array}$$

where ${\bar{g}}_{{\mu \nu }_{Ein}}\left(x\right)$ is the metric in Einstein frame while ${\bar{g}}_{{\mu \nu }_{Jor}}$ is the one in Jordan frame.

## 3. Rotating Black **Hole** Solutions

In this section, we analyze the rotating solutions, which satisfy the quadratic form of the field Equation (3). To execute it, we explore two cases separately:

i—the rotating case when N=4, and

ii—the rotating case when N>4.

i—the rotating case when N=4:

In this case, we apply the following coordinate transformations (from now on, in the case of N=4, we write the cosmological constant in the form of $\Lambda =-\frac{3}{\lambda^{2}}.$)

(14) $$\phi_{1}^{\prime }=\frac{a_{1}}{\lambda^{2}}\;t-\Xi \;\phi_{1},\;\;\;t^{\prime }=\Xi \;t-a_{1}\;\phi_{1},$$

where $a_{1}$ is the rotation parameter and Ξ is defined as

$$\Xi :=\sqrt{1+\frac{a_{1}{}^{2}}{\lambda^{2}}}.$$

With the transformation (14) to the metric (12), in the case of N=4, we acquire

(15) $$ds_{1}{}^{2}=-\left(\frac{\Xi^{2}\lambda^{2}h\left(r\right)-a_{1}{}^{2}r^{2}}{\lambda^{2}}\right)dt^{\prime }{}^{2}+\frac{dr^{2}}{h(r)}+r^{2}\left(\Xi^{2}d\phi_{1}^{\prime }{}^{2}+dz_{1}^{2}\right)-a_{1}{}^{2}h\left(r\right)d\phi_{1}^{\prime }{}^{2}+\frac{2\Xi a_{1}\left[r^{2}+\lambda h\left(r\right)\right]d\phi_{1}^{\prime }dt^{\prime }}{\lambda }\;,$$

where h(r) is given by Equation (9). It should be mentioned that, for the rotation parameter $a_{1}=0$, we return to the spacetime (12) with N=4.

For N>4, we apply the following coordinate transformations (from now on, in the case of N>4, we will write the cosmological constant, in the form of $\Lambda_{eff}=-\frac{\left(N-1)(N-2\right)}{\left(2\lambda_{1}{}^{2}\right)}.$):

(16) $$\phi_{i}^{\prime }=-\Xi \;\phi_{i}+\frac{a_{i}}{\lambda_{1}{}^{2}}\;t,\;\;\;t^{\prime }=\Xi \;t-\sum_{i=2}^{\ell }a_{i}\;\phi_{i},$$

where $a_{i}$, i>1 is the number of rotation parameters and Ξ is defined as

$$\Xi :=\sqrt{1+\sum_{i=1}^{\ell }\frac{a_{i}{}^{2}}{\lambda_{1}{}^{2}}}.$$

Applying the transformation (16) to the metric (12) in the case of N>4, we obtain

(17) $$\begin{array}{ccc} & & ds_{2}{}^{2}=-h\left(r\right){\left[\Xi dt^{\prime }-\sum_{i=2}^{\ell }a_{i}d\phi^{\prime }\right]}^{2}+\frac{dr^{2}}{h(r)}+\frac{r^{2}}{\lambda_{1}{}^{4}}\sum_{i=1}^{\ell }{\left[a_{i}dt^{\prime }-\Xi \lambda_{1}{}^{2}d{\phi^{\prime }}_{i}\right]}^{2}+r^{2}dz_{k}{}^{2}\\ & & -\frac{r^{2}}{\lambda_{1}{}^{2}}\sum_{i<j}^{\ell }{\left(a_{i}d{\phi^{\prime }}_{j}-a_{j}d{\phi^{\prime }}_{i}\right)}^{2}\;, \end{array}$$

where h(r) is given by Equation (10), $dz_{k}{}^{2}$ is the Euclidean metric in (N-*ℓ*-2)-dimensions and k=1,2⋯N−2.

Of note, the static configuration (12) can be recovered as a special case when the rotation parameters $a_{i}$ are equal to 0. It should stressed that, when the physical quantities $c_{1}$ for N=4 and $c_{2}$ for N>4 are vanishing, we obtain an odd AdS spacetime (it is notable that black hole solutions (15) and (17) are created by coordinate transformation and are new because they involve a rotation term that will be responsible for creating non-vanishing value of spatial momentum, as we will discuss in Section 5).

Finally, it should be stressed that coordinate transformations (14) are admitted locally, but not globally [103,104], because the compactified angular coordinate ϕ is mixed with the temporal coordinate *t* by coordinate transformations. This fact has been discussed in [105] by pointing out that, if the first Betti number for the manifold is a non-zero value, then the global diffeomorphisms, by which two spacetimes can be connected, do not exist, thus there is a new manifold that is globally parameterized by the rotation parameters $a_{i}$. The solution that is given by Equation (15) shows that the first Betti number is one of these solutions for the cylindrical or toroidal horizons. The same analysis can be applied to the coordinate transformation (16), for which the first Betti non-zero number is derived by the compactification of certain numbers of the angular coordinates in (N−2)-dimensional, $\phi_{i}$, to the submanifold of the solution. In this study, we call these coordinates rotation parameters of the solution.

## 4. Total Conserved Charge

We study the conserved quantities of the solutions found in the preceding section. For this purpose, we present the bases of the Einstein–Cartan geometry used for these calculations (because the Ricci scalar of solutions (15) and (17) is equal to −8Λ and $-8\Lambda_{eff}$, respectively, we are going to use the Komar formula to calculate the conserved quantities of the solutions that are derived in Section 3). The Lagrangian of this theory has the form [106] (the basic notations are given in Appendix A):

(18) $$V\left(\vartheta^{i},\;{R^{j}}_{k}\right):=-\frac{1}{2\kappa }\left(R^{ij}\wedge \eta_{ij}-2\Lambda \eta \right),$$

where $\vartheta^{i}$ is the coframe, $\eta_{ij}$ is the basis of two-form and $R^{ij}$ the Ricci that are one and two forms, respectively. Using the variational principle of Equation (18), we obtain [106,107]

(19) $$E_{i}:=-\frac{\partial V}{\partial \vartheta^{i}}=-\frac{1}{2\kappa }\left(R^{jk}\wedge \eta_{ijk}-2\Lambda \eta_{i}\right),\;\;B_{ij}:=-\frac{\partial V}{R_{ij}}=\frac{1}{2\kappa }\lambda_{ij},$$

where $B_{ij}$ and $E_{i}$ refer to the rotational gauge and energy-momentum, respectively. We can also define the following quantities

(20) $$E_{ij}:=-\vartheta_{[i}\wedge H_{j]}=0,\;\;\;\;\;\;\;\;H_{i}:=-\frac{\partial V}{\partial T^{i}}=0,$$

which correspond to spin and translation, respectively. The conserved quantity is represented in the form [106]

(21) $$\begin{array}{ccc} & & ȷ\left[\xi \right]=\frac{1}{2\kappa }d\left\{{}^{{}^{*}}\left[dk+\xi \rfloor \left(\vartheta^{i}\wedge T_{i}\right)\right]\right\},\;\mathrm{where}\;k=\xi_{i}\vartheta^{i},\;\mathrm{and}\;\xi^{i}=\xi \rfloor \vartheta^{i}, \end{array}$$

where ξ is a vector expressed as $\xi =\xi^{i}\partial_{i}$, with $\xi^{i}$ being *N* parameters and ∗ the Hodge duality. When the torsion one-form is vanishing, i.e., $T_{i}=0$, the total charge of Equation (21) reads

(22) $$Q\left[\xi \right]=\frac{1}{2\kappa }\int_{\partial S}{}^{*}dk.$$

This is the invariant conserved quantity [108,109,110,111].

We apply Equation (22) to the solutions (15) and (17). We calculate the necessary components for the case of N=4 and the co-frame is

(23) $$\begin{array}{ccc} & & {\vartheta^{{}^{{}^{\;\;\;\;}}}{}_{}{}_{}{}_{}}^{0}=\sqrt{h(r)}\left[\Xi dt^{\prime }-a_{1}d\phi_{1}^{\prime }\right],\;{\vartheta^{{}^{{}^{\;\;\;\;}}}{}_{}{}_{}{}_{}}^{\;1}=\frac{dr}{\sqrt{h(r)}},\;{\vartheta^{{}^{{}^{\;\;\;\;}}}{}_{}{}_{}{}_{}}^{\;2}=rdz_{1},\;{\vartheta^{{}^{{}^{\;\;\;\;}}}{}_{}{}_{}{}_{}}^{\;3}=r\Xi d\phi_{1}^{\prime }-\frac{ra_{1}}{\lambda }dt^{\prime }. \end{array}$$

With Equation (23) and Equation (21), we obtain

(24) $$k=\frac{\lambda^{4}h^{2}\left(r\right)\left(a_{1}\xi_{3}-\Xi \xi_{0}\right)\left(\Xi dt^{\prime }-a_{1}d\phi_{1}^{\prime }\right)+\lambda^{4}\xi_{1}dr+r^{2}h\left(r\right)\left[\lambda^{4}\xi_{2}dz_{1}+\left(\lambda^{4}\Xi^{2}\xi_{3}-\lambda^{2}a_{1}\Xi \xi_{0}\right)d\phi_{1}^{\prime }+a_{1}\left(a_{1}\xi_{0}-\lambda^{2}\Xi \xi_{3}\right)dt^{\prime }\right]}{\lambda^{4}h\left(r\right)}.$$

The total derivative of Equation (24) gives

(25) $$\begin{array}{c} dk=\frac{1}{\lambda^{4}h\left(r\right)}[\{h^{\prime }\left(r\right)\left\{\lambda^{4}\Xi \left(a_{1}\xi_{3}-\Xi \xi_{0}\right)h\left(r\right)+r^{2}a_{1}\left(\lambda^{2}\Xi \xi_{3}-a_{1}\xi_{0}\right)\right\}-ra_{1}\left(\lambda^{2}\Xi \xi_{3}-a_{1}\xi_{0}\right)\left[rh^{\prime }\left(r\right)+2h\left(r\right)\right]\}\left(dr\wedge dt^{\prime }\right)\\ +2\lambda^{4}rh\left(r\right)\xi_{2}\left(dr\wedge dz_{1}\right)-\lambda^{2}\{h^{\prime }\left(r\right)\left\{\lambda^{2}a_{1}\left(a_{1}\xi_{3}-\Xi \xi_{0}\right)h\left(r\right)+r^{2}\Xi \left(\lambda^{2}\Xi \xi_{3}-a_{1}\xi_{0}\right)\right\}-r\Xi \left(\lambda^{2}\Xi \xi_{3}-a_{1}\xi_{0}\right)\left[rh^{\prime }\left(r\right)+2h\left(r\right)\right]\}\\ \times (dr\wedge d\phi_{1}^{\prime })]. \end{array}$$

Using Equation (23), we obtain

(26) $$dt^{\prime }=\frac{1}{r}\left(\frac{\vartheta^{0}r\Xi }{\sqrt{h(r)}}+\frac{\vartheta^{3}a_{1}}{\lambda^{2}}\right),\;\;d\phi_{1}^{\prime }=\frac{1}{r}\left(\vartheta^{3}\Xi +\frac{\vartheta^{0}ra_{1}}{\lambda^{2}\sqrt{h(r)}}\right),\;dr=\vartheta^{1}\sqrt{h(r)},\;dz_{1}=\frac{\vartheta^{2}}{r}.$$

By combining Equation (25) with Equation (26) and Equation (22) and operating the Hodge-dual to dk, we acquire the following forms for the total conserved charge

(27) $$Q\left[\xi_{t}^{\prime }\right]=\frac{\Xi^{2}}{\lambda^{2}}\left(M-4r^{3}\right),\;\;Q\left[\xi_{r}\right]=Q\left[\xi_{z_{k}}\right]=0,\;Q\left[\xi_{\phi_{1}^{\prime }}\right]=\frac{a_{1}\Xi }{\lambda^{2}}\left(M-4r^{3}\right),$$

where $M=-c_{1}$. Throughout the same calculations for the case of N>4, with Equation (17), we find

(28) $$\begin{array}{ccc} & & {\vartheta^{{}^{{}^{\;\;\;\;}}}{}_{}{}_{}{}_{}}^{0}=\sqrt{h(r)}\left[\Xi dt^{\prime }-\sum_{i=1}^{\ell }a_{i}d\phi_{i}^{\prime }\right],\;{\vartheta^{{}^{{}^{\;\;\;\;}}}{}_{}{}_{}{}_{}}^{\;1}=\frac{dr}{\sqrt{h(r)}},\;{\vartheta^{{}^{{}^{\;\;\;\;}}}{}_{}{}_{}{}_{}}^{\;z_{1}}=rdz_{1},\;{\vartheta^{{}^{{}^{\;\;\;\;}}}{}_{}{}_{}{}_{}}^{\;z_{2}}=rdz_{2},\cdots \;{\vartheta^{{}^{{}^{\;\;\;\;}}}{}_{}{}_{}{}_{}}^{\;z_{N-\ell -2}}=rdz_{N-\ell -2},\\ & & {\vartheta^{{}^{{}^{\;\;\;\;}}}{}_{}{}_{}{}_{}}^{\;\phi_{i}^{\prime }}=r\Xi d\phi_{i}^{\prime }-\frac{ra_{i}}{\lambda^{2}}dt^{\prime }, \end{array}$$

where i=2⋯⋯ℓ. By substituting Equation (28) into Equation (22), we acquire

(29) $$\begin{array}{ccc} & & k=\frac{1}{\lambda^{4}h\left(r\right)}[h^{2}\left(r\right)\lambda^{4}\left(\sum_{i=1}^{\ell }a_{i}\xi_{i+k+1}-\Xi \xi_{0}\right)\left(\Xi dt^{\prime }-\sum_{i=1}^{\ell }a_{i}d\phi_{i}^{\prime }\right)+\lambda^{4}\xi_{1}dr+r^{2}h\left(r\right)(\lambda^{4}\sum_{i=1}^{k}\xi_{i+1}dz_{i}\\ & & +\sum_{i=1}^{\ell }\left(\lambda^{4}\Xi^{2}\xi_{i+k+1}-\lambda^{2}a_{i}\Xi \xi_{0}\right)d\phi_{i}^{\prime }+\sum_{i=1}^{\ell }\left(a_{i}{}^{2}\xi_{0}-\lambda^{2}\Xi a_{i}\xi_{i+k+1}\right)dt^{\prime })]. \end{array}$$

The total derivative of Equation (29) yields

(30) $$\begin{array}{ccc} & & dk=\frac{1}{\lambda_{1}{}^{4}h\left(r\right)}[\{h^{\prime }\left(r\right)\left[\lambda_{1}{}^{4}\Xi \left(\sum_{i=1}^{\ell }a_{i}\xi_{i+k+1}-\Xi \xi_{0}\right)h\left(r\right)+r^{2}\left(\lambda_{1}{}^{2}\Xi \sum_{i=1}^{\ell }a_{i}\xi_{i+k+1}-{\left(\sum_{i=1}^{\ell }a_{i}\right)}^{2}\xi_{0}\right)\right]-r(\lambda_{1}{}^{2}\Xi \sum_{i=1}^{\ell }a_{i}\xi_{i+k+1}\\ & & -{\left(\sum_{i=1}^{\ell }a_{i}\right)}^{2}\xi_{0})\left[rh^{\prime }\left(r\right)+2h\left(r\right)\right]\}\left(dr\wedge dt^{\prime }\right)+2\lambda_{1}{}^{4}rh\left(r\right)\sum_{i=1}^{k}\xi_{i+1}\left(dr\wedge dz_{i}\right)-\lambda_{1}{}^{2}\sum_{i=1}^{\ell }\left(dr\wedge d\phi_{i}^{\prime }\right)\{h^{\prime }\left(r\right)\{\lambda_{1}{}^{2}a_{i}(\sum_{j=1}^{\ell }a_{j}\xi_{j+k+1}\\ & & -\Xi \xi_{0})h\left(r\right)+r^{2}\Xi \left(\lambda_{1}{}^{2}\Xi \xi_{i+k+1}-a_{i}\xi_{0}\right)\}-r\Xi \left(\lambda_{1}{}^{2}\Xi \xi_{i+k+1}-a_{i}\xi_{0}\right)\left[rh^{\prime }\left(r\right)+2h\left(r\right)\right]\}]. \end{array}$$

We calculate the inverse of Equation (28), as we have done in the four-dimensional case. By combining the results and Equation (30) and while using the Hodge-dual, we find that the conservation of N>4 of Equation (17) is represented as

(31) $$Q\left[\xi_{t}\right]=\frac{\Omega_{D-1}h^{\prime }\left(r\right)}{16\pi \lambda_{1}{}^{2}},\;\;Q\left[\xi_{r}\right]=Q\left[\xi_{z_{i}}\right]=0,\;Q\left[\xi_{\phi_{i}}\right]=\frac{a_{i}h^{\prime }\left(r\right)\Omega_{D-1}}{16\pi \lambda_{1}{}^{2}},$$

where h(r), in the case of four-dimensions, is given by Equation (9) and $h^{\prime }\left(r\right)=\frac{dh(r)}{dr}$ and in the case N>4h(r) is given by (10).

Equations (27) and (31) show that the conserved quantities of spacetimes (15) and (17) are divergent. Thus, regularization is necessary for Equation (22).

## 5. Regularization with Relocalization for the Conserved Charge

It is observed that, for the general coordinate and local Lorentz transformations, Equation (18) is invariant. However, in [106], it has been indicated that there is one more vagueness in terms of the definition for the conserved quantities, rather than the diffeomorphism and local Lorentz freedom. This occurs because the relocalization in terms of the momenta of the gravitational field can always be allowed by field equations. Relocalization is generated from the change of the Lagrangian for the gravitational field in terms of the total derivative, which is described as

(32) $$V^{\prime }=V+d\Phi ,\;\Phi =\Phi \left({\vartheta^{{}^{{}^{\;\;\;\;}}}{}_{}{}_{}{}_{}}^{\alpha },{\Gamma_{\alpha }}^{\beta },T^{\alpha },{R_{\alpha }}^{\beta }\right).$$

The second term in the right-hand-side of Equation (32) is responsible for the change of the boundary part for the action only; thus, the field equations are left unaltered [106]. For the relocalization method, the conserved charge is [106]

(33) $$J\left[\xi \right]=-\frac{\lambda^{2}}{4\kappa }\int_{\partial S}\eta_{\alpha \beta \mu \nu }\Xi^{\alpha \beta }W^{\mu \nu }.$$

Here, $W^{\mu \nu }$ is the Weyl two-form, as given by

(34) $$W^{\alpha \beta }=\frac{1}{2}{C_{\mu \nu }}^{\alpha \beta }\vartheta^{\mu }\wedge \vartheta^{\nu },$$

where ${C_{\mu \nu }}^{\alpha \beta }={h_{\mu }}^{i}{h_{\nu }}^{j}{h^{\alpha }}_{k}{h^{\beta }}_{l}{C_{ij}}^{kl}$ is the Weyl tensor, and $\Xi^{\alpha \beta }$ is represented as (In [106,112,113,114], explanations for the way of deriving Equation (33) are provided.)

(35) $$\Xi_{\alpha \beta }=\frac{1}{2}e_{\beta }\rfloor e_{\alpha }\rfloor dk.$$

It is known that, for the coordinate and local Lorentz transformations, the conserved currents J[ξ] do not change. The vector field ξ on the manifold of the spacetime is associated with the currents J[ξ]. Equation (33) is used to analyze the conserved quantities in terms of spacetimes (15) and (17).

In the case of N=4, with the metric spacetime (15), we examine the components of Equation (33). The non-zero components in terms of $\Xi^{\alpha \beta }$ read (in Appendix B, the non-zero components of the Weyl tensor are described.)

(36) $$\Xi_{01}=-\frac{\left[\Xi \xi_{0}+a_{1}\xi_{3}\right]\left[c_{1}\lambda^{2}-4r^{3}\right]}{2r^{2}\lambda^{2}},\;\;\;\Xi_{13}=-\frac{\left[a_{1}\xi_{0}-\Xi \xi_{3}\lambda^{2}\right]\sqrt{h(r)}}{\lambda^{2}},$$

Using Equations (33) and (36), we obtain

(37) $$\eta_{\alpha \beta \mu \nu }\Xi^{\alpha \beta }W^{\mu \nu }≅-\frac{4c_{1}\left(\left[a_{1}{}^{2}+2\lambda^{2}\Xi^{2}\right]\xi_{0}+a_{1}\Xi \lambda^{2}\xi_{3}\right)}{\lambda^{4}}+O\left(\frac{1}{r^{3}}\right).$$

The substitution of Equation (37) into Equation (33) leads to

(38) $$J\left[\xi_{t}\right]=M\left[3\Xi^{2}-1\right],\;J\left[\xi_{r}\right]=J\left[\xi_{\theta }\right]=0,\;J\left[\xi_{\phi_{1}}\right]=Ma_{1}\Xi ,$$

which is consistent with the result presented in [91,115].

Throughout the same procedure for the metric spacetime (17), we acquire the following non-zero components in terms of $\Xi^{\alpha \beta }$

(39) $$\begin{array}{ccc} & & \Xi_{1t}=\left[\sum_{i=0}^{\ell }a_{1+i}\xi_{n-k+i}-\Xi \xi_{0}\right]h^{\prime }\left(r\right),\;\Xi_{1n-k+i}=-\frac{2\left[a_{1+i}\xi_{0}-\Xi \xi_{n-k+i}\lambda_{1}{}^{2}\right]\sqrt{h(r)}}{\lambda_{1}{}^{2}}, \end{array}$$

where h(r) is given by Equation (10). Using Equation (33), we have

(40) $$\eta_{\alpha \beta \mu \nu }\Xi^{\alpha \beta }W^{\mu \nu }≅\frac{4c_{1}\left(\left[\sum_{i=0}^{\ell }a_{i}{}^{2}+\left(n-2\right)\lambda_{1}{}^{2}\Xi^{2}\right]\xi_{0}+\sum_{i=0}^{\ell }a_{i}\Xi \lambda_{1}{}^{2}\xi_{3}\right)}{\lambda_{1}{}^{2}}+O\left(\frac{1}{r^{6}}\right).$$

By combining Equations (33) and (40), we obtain

(41) $$J\left[\xi_{t}\right]=M\left[\left(n-1\right)\Xi^{2}-1\right]\xi_{0},\;J\left[\xi_{r}\right]=J\left[\xi_{\theta }\right]=0,\;J\left[\xi_{\phi_{1+i}}\right]=Ma_{1+i}\Xi \xi_{n-\ell +i},$$

where i=1,2⋯ℓ−1 and we have put $c_{2}=-M$. Equation (41) is compatible with what is derived in [115].

## 6. Thermodynamics for Black Holes

In this section, we describe the thermodynamic quantities (e.g., temperature, entropy, and heat capacity) of the black hole solutions (9) and (10). For this, we define the Hawking temperature as [91,116,117,118]:

(42) $$T_{h}=\frac{h^{\prime }\left(r_{h}\right)}{4\pi }.$$

**Figure 1:**
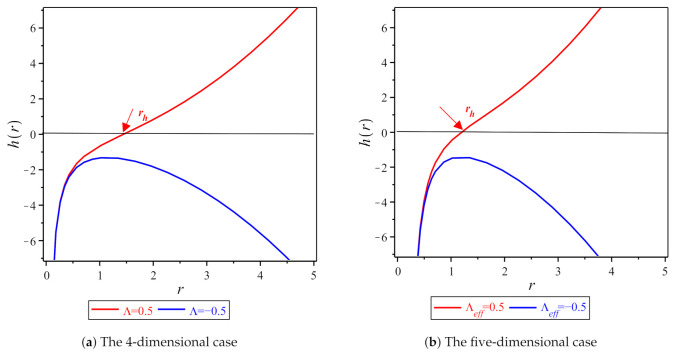
The function *h*(*r*) vs. the radial coordinate *r* for (**a**) *N* = 4, $c_{1}=-1$. (**b**) *N* = 5, $c_{2}=-1$ (all of the figures are reproduced using the Maple software 16).

Using Equations (9) and (10) in Equation (42), we obtain

(43) $$T_{h}=\frac{r_{h}\Lambda }{4\pi },\;\mathrm{when}\;N=4,\;T_{h}=\frac{\left(N-1\right)r_{h}\Lambda_{eff}}{4\pi },\;\mathrm{when}\;N>4,$$

where $r_{h}$ is the largest root of the function h(r) that is given by Equations (9) and (10), respectively. The relation between the function h(r) and radial coordinate *r* for black hole solutions (9) and (10) is plotted in Figure 1a,b, which show that we have an outer event horizon for the positive cosmological constant.

**Figure 2:**
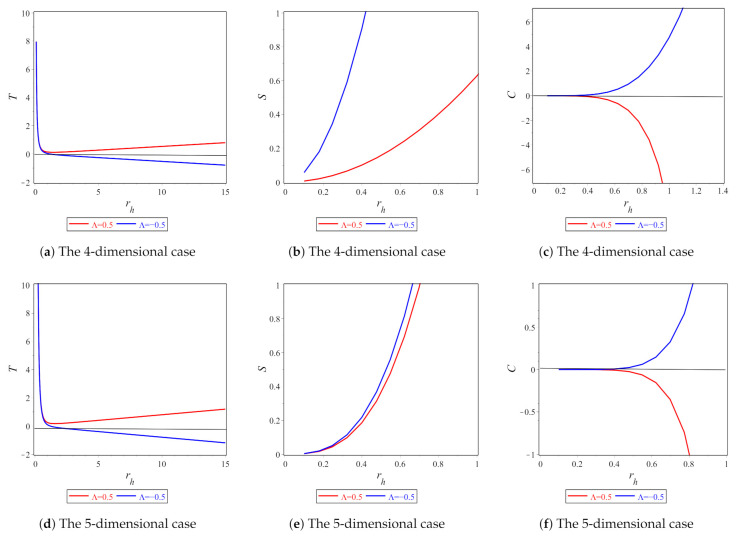
Horizon $r_{h}$ vs. (**a**,**d**) Hawking temperature (**b**,**e**); entropy (**c**,**f**) heat capacity for the four-dimensional and five-dimensional cases, respectively. In these figures, we take b=0.1 when N=4 and b=0.01 when N=5.

Additionally, in Figure 2a,d, we plot the behavior of temperature vs. the horizon for N=4 and N=5. The abovementioned figures show that we have a positive temperature for positive Λ and vice versa, i.e., a negative temperature for negative Λ. This negative Hawking temperature is responsible for forming an ultracold black hole. Davies [119] has approved this result, who has shown that there is no logic in preventing a black hole temperature from having a negative value to switch it to a naked singularity. In fact, this is the case presented in Figure 2a,d. The case of an ultracold black hole can be explained by the existence of a phantom energy field [120]. Moreover, it has been shown that the negative nature of temperature is related to quantum properties [121].

In order to investigate the thermodynamic quantities in the context of black hole solutions (9) and (10), we set the constraint $h(r_{h})=0$, which gives

(44) $$\begin{array}{ccc} & & {M_{h}}_{{}_{{}_{{}_{{}_{Equation\;(9)}}}}}=\frac{2r_{h}{}^{3}\Lambda }{3}\;\mathrm{for}\;N=4,\;{M_{h}}_{{}_{{}_{{}_{{}_{Equation\;(10)}}}}}=r_{h}{}^{N-1}\Lambda \;\mathrm{for}\;,\;\mathrm{when}\;N>4. \end{array}$$

Now, we will briefly discuss the entropy of black hole in f(R) gravity. For this, we use the arguments that are presented in [122]. From the Noether method, which was used to calculate the entropy associated with black holes in the f(R) theory that have a constant Ricci scalar, one can obatin [98]

(45) $$S=\frac{1}{4}Af_{R}\left(R\right){|}_{r=r_{h}},$$

where A is the area of the event horizon. Using Equations (9) and (10), we obtain the entropy as

(46) $$S=\pi r_{h}{}^{2}\left(1-16b\Lambda \right),\;\mathrm{when}\;N=4,\;S=\frac{\Omega_{N-2}r_{h}{}^{N-2}\left(1-16b\Lambda_{eff}\right)}{4\pi },\;\mathrm{when}\;N>4$$

where $\Omega_{N-2}$ denotes the volume of the unit (N−2)-sphere. We plot the behavior of entropy in Figure 2b,e. It should be stressed that, for positive entropy, especially for a positive cosmological constant, the dimensional parameter *b* must be $b<\frac{1}{16\Lambda }$ when N=4 and $b<\frac{1}{16\Lambda_{eff}}$ for N>4. This puts a constraint on the parameter *b* [123].

Finally, it is known that there are several ways to study the stability of a black hole [124]: among these approaches is the thermodynamic stability, which is related to the sign of its heat capacity $C_{h}$. Now, we are going to analyze the thermal stability of black hole solutions via the behavior of their heat capacities [125,126,127]

(47) $$C_{h}=\frac{\partial M_{h}}{\partial r_{h}}{\left(\frac{\partial T}{\partial r_{h}}\right)}^{-1}.$$

If the heat capacity $C_{h}>0$ ($C_{h}<0$), then the black hole is thermodynamically stable (unstable). Thus, a black hole with a negative heat capacity is thermally unstable. Using Equations (42) and (44) in (47), we obtain

(48) $$C_{h}=4\pi r_{h}{}^{2},\;\mathrm{for}\;N=4;\;C_{h}=4\pi r_{h}{}^{N-2},\;\mathrm{for}\;N>4.$$

The behavior of heat capacity is plotted in Figure 2b,e, which show that the black hole solutions (9) and (10) are stable when Λ<0. The case of negative temperature has been discussed in [97,121].

## 7. Summary and Discussion

Recently, without introducing any exotic matter, extended theory of gravity, F(R), has been considered to be an alternative approach for explaining the galactic rotation curves and the cosmic acceleration [39,44,60]. The approach results from effective theory aimed to deal with quantum fields in curved space-time at ultraviolet scales that give rise to additional contributions with respect to GR also at infrared scales: in this perspective, galactic, extra-galactic, and cosmological scales can be affected by these gravitational corrections without requiring large amounts of unknown material dark components. In the framework of f(R), one may consider that the gravitational interaction acts differently at different scales, while the results of GR at Solar System scales are preserved. In other words, GR is a particular case of a more extended class of f(R) gravitational theory. From a conceptual viewpoint, there is no a priori reason to restrict the gravitational Lagrangian to a linear function of the Ricci scalar minimally coupled to matter.

In this study, we derived *N*-dimension, N>4, black hole solutions in f(R) gravitation theory. We applied a spacetime (which possesses a *k*-dimension Euclidean metric, *ℓ*-dimension angular coordinates, and one unknown function of the radial coordinate) to the gravitational field equations in the f(R) gravity with the quadratic form $f\left(R\right)=R+bR^{2}$. The resulting differential equations are solved exactly without any assumption and general solutions for N=4 and N>4. These solutions are classified, as follows:

(i) The solution of N=4 is completely identical to GR and it gives a planar black hole spacetime that is a singular one.

(ii) The solutions with N>4 are affected by the higher curvature order, i.e., the solutions contain the dimensional parameter *b*. Generally, the solutions in the case of N>4 cannot reduce to the GR solutions, because the parameter *b* is not allowed to go to 0. Of note, metric (6) satisfies $g_{tt}g_{rr}=1$, which, of course, is not the general one. We would like to emphasize that, even if we use two different unknown functions, the solutions of the resulting differential equation will give $g_{tt}g_{rr}=1$ after some re-scaling.

To construct rotating black hole solutions, we applied a coordinate transformation that relates the temporal coordinate and the rotating one in the case of N=4 and between the temporal and angular-coordinates in the case of N>4 and derived the solutions of rotating black hole that satisfy gravitational field equations for $f\left(R\right)=R+bR^{2}$. The topology of the output solution in the case of N=4 is a cylindrical spacetime with $R\times S^{1}$ and $0\leq \phi_{1}<2\pi$ and $-\infty <z_{1}<\infty$; in the case of N>4, it is $0\leq \phi_{\ell }<2\pi$ and $-\infty <z_{k}<\infty$ in the case of N>4.

We studied the physics of the rotating black hole solutions by calculating their conserved quantities using Komar [108]. This method provides divergent quantities of energy and angular momentum for the two cases of N=4 and N>4. Thus, we used the regularization method to obtain the energy-momentum and angular one with their finite values. Relocalization is the regularization method used in this study, which is created from the change of the Lagrangian for the gravitational field in terms of the total derivative. From the method, the representations for the energy-momentum and angular one were acquired. It was also confirmed that these results were consistent with those that were derived in [91,104].

Finally, we derived the entropies of black hole solutions (9) and (10), and showed that they were not proportional to the area of horizons because of the existence of the $f_{R}$ term that is not trivial in our study [91]. From these calculations, we put a constraint on the dimensional parameter *b* to get positive entropy. Additionally, we studied heat capacity for the cases of N=4 and N>4. We showed that the system is thermally stable, for Λ>0 and $\Lambda_{eff}>0$, in both cases, as shown in Figure 2c. Here, we emphasize that the case of N=4 completely coincides with GR; however, if one considers the case $f\left(R\right)=R-2\alpha \sqrt{R}$, the situation is different [97] and one obtained a new solution in the case of N=4. A detailed analysis of this solution regarding thermodynamic analysis was performed [97].

## Data Availability

Data sharing not applicable.

## Appendix A. Symbols Used in the Calculations of Conserved Quantities

Indices k,l,⋯ are the coframe indices and γ, δ, ⋯ are the coordinate ones. The wedge product is represented by ∧ and the interior product is given by ξ⌋Ψ. The coframe $\vartheta^{i}$ is defined as $\vartheta^{i}={e^{i}}_{\mu }dx^{\mu }$ and the frame $e_{i}$ is defined as $e_{i}={e_{i}}^{\mu }\partial_{\mu }$ with ${e^{i}}_{\mu }$ and ${e_{i}}^{\mu }$ being the covariant of the vielbein and its contravariant, respectively. The volume is expressed by $\eta :=\vartheta^{\hat{0}}\wedge \vartheta^{\hat{1}}\wedge \vartheta^{\hat{2}}\wedge \vartheta^{\hat{3}}$. In addition, we describe

$$\eta_{i}:=e_{i}\rfloor \eta =\;\frac{1}{3!}\;\epsilon_{ijkl}\;\vartheta^{j}\wedge \vartheta^{k}\wedge \vartheta^{l},$$

where $\epsilon_{ijkl}$ is completely antisymmetric.

## Appendix B. Non-Zero Components for the Christoffel Symbols of the Second Kind and Ricci Curvature Tensor

Using Equation (6), we find the non-zero components for the Christoffel symbols of the second kind and Ricci curvature tensor

$$\begin{array}{ccc} & & \Gamma^{t}{}_{t\;t}=-\Gamma^{r}{}_{r\;r}=\frac{h^{\prime }}{2h},\;\;\Gamma^{t}{}_{r\;t}=\frac{hh^{\prime }}{2},\\ & & \Gamma^{r}{}_{\phi_{1}\;\phi_{1}}=\Gamma^{r}{}_{\phi_{2}\;\phi_{2}}\cdots \cdots \Gamma^{r}{}_{\phi_{N-\ell }\;\phi_{N-\ell }}=\Gamma^{r}{}_{z_{1}\;z_{1}}=\Gamma^{r}{}_{z_{2}\;z_{2}}\cdots \cdots \Gamma^{r}{}_{z_{N-\ell -2}\;z_{N-\ell -2}}=-rh,\\ & & \Gamma^{\phi_{1}}{}_{r\;\phi_{1}}=\Gamma^{\phi_{2}}{}_{r\;\phi_{2}}\cdots \cdots \Gamma^{\phi_{N-\ell }}{}_{r\;\phi_{N-\ell }}=\Gamma^{z_{1}}{}_{r\;z_{1}}=\Gamma^{z_{2}}{}_{r\;z_{2}}\cdots \cdots \Gamma^{z_{N-\ell -2}}{}_{r\;\phi_{N-\ell -2}}=\frac{1}{r}. \end{array}$$

$$\begin{array}{ccc} & & R_{t\;r\;t\;r}=\frac{h^{\prime \prime }}{2},\;R_{t\;\phi_{1}\;t\;\phi_{1}}=R_{t\;\phi_{2}\;t\;\phi_{2}}=\cdots \cdots R_{t\;\phi_{N-\ell }\;t\;\phi_{N-\ell }}=R_{t\;z_{1}\;t\;z_{1}}=R_{t\;z_{2}\;t\;z_{2}}=\cdots \cdots R_{t\;z_{N-\ell -2}\;t\;z_{N-\ell -2}}=\frac{r\;hh^{\prime }}{2},\\ & & R_{r\;\phi_{1}\;r\;\phi_{1}}=R_{r\;\phi_{2}\;r\;\phi_{2}}=\cdots \cdots R_{r\;\phi_{N-\ell -2}\;r\;\phi_{N-\ell -2}}=R_{r\;z_{1}\;r\;z_{1}}=R_{r\;z_{2}\;r\;z_{2}}=\cdots \cdots R_{r\;z_{N-2}\;r\;z_{N-2}}=-\frac{rh^{\prime }}{2h},\\ & & R_{\phi_{1}\;\phi_{2}\;\phi_{1}\;\phi_{2}}=R_{\phi_{1}\;\phi_{3}\;\phi_{1}\;\phi_{3}}=\cdots \cdots R_{\phi_{1}\;\phi_{N-\ell }\;\phi_{1}\;\phi_{N-\ell }}=R_{\phi_{2}\;\phi_{3}\;\phi_{2}\;\phi_{3}}=R_{\phi_{2}\;\phi_{4}\;\phi_{2}\;\phi_{4}}\cdots \cdots =\\ & & R_{\phi_{2}\;\phi_{N-\ell }\;\phi_{2}\;\phi_{N-\ell }}\cdots \cdots R_{\phi_{N-\ell -1}\;\phi_{N-\ell }\;\phi_{N-\ell -1}\;\phi_{N-\ell }}=R_{z_{1}\;z_{2}\;z_{1}\;z_{2}}=R_{z_{1}\;z_{3}\;z_{1}\;z_{3}}=\cdots \cdots R_{z_{1}\;z_{N-\ell -2}\;z_{1}\;z_{N-\ell -2}}=\\ & & R_{z_{2}\;z_{N-\ell -2}\;z_{2}\;z_{N-\ell -2}}\cdots \cdots R_{z_{N-\ell -3}\;z_{N-\ell -2}\;z_{N-\ell -3}\;z_{N-\ell -2}}=-r^{2}h, \end{array}$$
